# UVC radiation as an effective disinfectant method to inactivate human papillomaviruses

**DOI:** 10.1371/journal.pone.0187377

**Published:** 2017-10-31

**Authors:** Craig Meyers, Janice Milici, Richard Robison

**Affiliations:** 1 Department of Microbiology and Immunology, Pennsylvania State College of Medicine, Hershey, Pennsylvania, United States of America; 2 Department of Microbiology & Molecular Biology, Brigham Young University, Provo, Utah, United States of America; Georgetown University, UNITED STATES

## Abstract

Endocavitary ultrasound probes are part of a commonly used procedure in the clinical arena. The cavities examined, vaginal canal and cervix, anal canal, and oral cavity are all areas commonly infected with the human papillomavirus (HPV), thus making them susceptible to contamination by HPV. It has been demonstrated that these probes can remain contaminated with high-risk HPV even when approved disinfection protocols have been performed. we have previously shown that HPV is resistant to some high-level disinfectant (HLD). In our present study we analyzed efficacy of using high-level ultra-violet C (UVC) radiation against HPV16 and HPV18 using a hard-surface carrier test. Stocks of infectious authentic HPV16 and HPV18 virions were dried onto carriers with a 5% (v/v) protein soil or 4ppm hard water. Efficacy testing were performed with the automated device, Antigermix S1 device (UVC radiation at 253.7nm) and 0.55% OPA in quadruplicate with matched input, neutralization, and cytotoxicity controls. Hypochlorite was included as a positive control for viral deactivation. Infectivity was determined by the abundance (qRT-PCR) of the spliced E1^E4 transcript in infected recipient cells. The automated Antigermix S1 device showed excellent efficacy against HPV16 and HPV18 whereas OPA showed minimal efficacy. While HPV is highly resistant to OPA, high-level UVC radiation offers an effective disinfection practice for ultrasound probes. Our results suggest that healthcare facilities using endocavitary ultrasound probes need to strongly consider disinfection methods that are effective against HPV.

## Introduction

Human papillomaviruses (HPV) are responsible for over 5% of human cancers worldwide. Nearly all cases of cervical cancer are the result of HPV infection, as well as a significant number of anogenital and oropharyngeal cancers. While HPV is commonly known to be transmitted by sexual contact, the public is unaware of the risk of HPV infection through non-sexual transmission [1]. Often the potential for non-sexual infection by HPV is ignored or dismissed by healthcare professionals. While the actual number of non-sexual transmission of HPV is unknown, and will be difficult to determine, there is a risk for non-sexual transmission. Several studies have provided evidence for a route of vertical transmission HPV from mother or child [2–8]. A couple of studies detected abundant HPV DNA on the fingertips of individuals with anogenital infections, providing a potentially important vehicle for self-inoculation and inoculation of partners [9, 10]. The potential for non-sexual HPV infection HPV can remain infectious in a typical room environment for days further increases the possibility for non-sexual transmission [11, 12]. Until recently not a lot has been done to determine the efficacy of common hospital disinfectants to inactivate HPV.

The two pathways, direct host-to-host and non-direct fomite infection pathways, require the ability to survive great environmental pressures, the characteristics of these pressures are often different. The HPV life cycle including the assembly of infectious particles has a strict requirement for differentiating epithelium. HPV particle assembly is an intricate process, which includes the formation of stabilizing disulfide bonds [13–20]. The disulfide bonds form within the oxidizing environment of the host epithelium's cornified layer, which is important for the final stages in the maturation process of the infectious HPV virion [17]. Our laboratory and others have shown that the inter-pentameric disulfide bonding of three conserved cysteines in the major capsid protein L1 were necessary to guide the assembly process to full virion maturation [16, 21–23]. An additional three cysteine residues in the L! capsid protein have also been suggested to play a role in the assembly and maturation process [24].

Hospital-acquired, nosocomial or iatrogenic, infections are well-established routes for many infectious agents including viruses. It has been reported that transvaginal ultrasound probes can remain contaminated with HPV even when proper hospital disinfectant reprocessing procedures are followed [25–28]. Use of these probes is a common practice in emergency and gynecology departments of healthcare facilities. Sensitivity to damage by autoclaving and certain chemical disinfection procedures, puts limitations on the disinfectant procedures that are available. Probe sheaths and condoms have been used in an attempt to increase protection of the actual probe from contamination, but these coverings can significant perforation frequencies [29–32]. In one study even when visual observations could detect perforations or breaks in the probe covers there was still evidence of HPV contamination on the probe [25]. In past studies glutaraldehydes (GTA) and ortho-phthalaldehydes (OPA) had no effect on HPV infectivity [33]. These results were important as these two disinfectants are commonly used because they are classified as high-level disinfectants and considered sterilants in healthcare facilities. A follow up study showed that high concentration sonicated hydrogen peroxide was an effective disinfection to deactivate HPV on ultrasound probes [34]. We now report that using the ANTIGERMIX device we tested the effectiveness of high-level UVC to deactivate HPV16 and HPV18.

## Materials and methods

### Study design

In this study we used the hard surface carrier test method based on the ASTM E1053-11 standard test method suitable for assessing virucidal activity on non-porous surfaces [35]. This standard meets the Environmental Protection Agency (EPA) efficacy data requirements for virucides, which are in turn referenced by the FDA for 510(k) submissions for high-level disinfectants [36].

### Cell culture and virus production

The HaCaT cell line was maintained in DMEM supplemented with 10% FBS, 0.025 mg/ml Gentamicin, and 0.11 mg/ml sodium pyruvate. Primary human keratinocytes were isolated from newborn foreskin circumcision as previously described [37]. Primary keratinocytes were maintained in 154 medium supplemented with Human Keratinocyte Growth Supplement Kit (Cascade Biologics, Inc., Portland, OR). Immortalized keratinocytes stably maintaining HPV episomes were cultured in E-medium with J2-3T3 feeder cells and grown in raft culture to produce virus as previously described [37, 38]. Mature virus particles were harvested from tissues after 20 days. Rafts were harvested and virus was isolated by homogenization in phosphate buffer (5mM Na-phosphate, pH 8, 2mM MgCl2) as previously described [37]. All virus preparations for disinfectant treatment and infectivity assays were treated with benzonase (375 U) at 37°C for one hour to remove any un-encapsidated viral genomes. Samples were adjusted to 1M NaCl and centrifuged at 4°C for 10 min at 10,500 rpm to remove cellular debris [37].

The use of human foreskin keratinocyte tissues to develop cell lines for these studies was approved by the Institutional Review Board at the Pennsylvania State University College of Medicine and by the Institutional Review Board at Pinnacle Health Hospitals. Discarded tissues were exempt from needing informed patient consent. Informed consent was waived by both Institutional Review Boards. No identifiers were attached to any tissue samples.

### Virus titers

Viral genomes were released using 10ml of a virus preparation resuspended in a 200ml HIRT DNA extraction buffer (400mM NaCl/10mM Tris-HCl, pH 7.4/10mM EDTA, pH 8.0), with 2ml 20mg/ml Proteinase K, and 10ml 10% SDS for 2 hr at 37°C. Phenol-chloroform extraction followed by ethanol precipitation and re-suspended in 20ml TE purified the DNA. Viral titers were then determined using a qPCR-based DNA encapsidation assay utilizing a Qiagen Quantitect SYBR Green PCR Kit. Amplification of the viral genome target was performed using previously described HPV E2 open reading frame primers against a standard curve of 10-fold serial dilutions from 10^8^ to 10^4^ copies per ml [37].

### Infections, neutralizations, and inhibition assays

HaCaT cells were seeded at 50,000 cells per well in 24-well plates, 2 days prior to infection. Virus and media were in a total volume of 500ml prior to addition to the cells. A multiplicity of infection (MOI) of 10 was used unless otherwise noted. Virus was incubated with the cells for 48 hr at 37°C and mRNA were harvested using a Qiagen RNAeasy Kit.

### Carrier preparation

Carriers were 50-3mm circular discs made of acrylonitrile butadiene styrene, a plastic used in ultrasound transducer construction. Carriers were prepared by soaking in 10% hydrogen peroxide for 15min, neutralization in sterile water containing 200U/ml of catalase for 10min and rinsing in sterile water for 10min before being dried in a sterile petri dish. An organic load (soil) of 5% BSA or 4ppm CaCO_3_ (hard water) was added to the virus suspension and 200ml of this was spread onto a single carrier side with a sterile pipette tip. The inoculated carriers were allowed to dry in a laminar flow cabinet for 30 min or until dry.

### Disinfectants

The Antigermix automated disinfection unit was used to test its efficacy to inactivate authentic HPV16 and HPV18. The carriers containing HPV were hung in the Antigermix device. The devise uses a strong exposure to ultra-violet radiation in a 90 sec preprogrammed cycle according to the manufacturer’s instructions. As a negative control for disinfection we included liquid OPA at the manufacturers recommended concentration of 0.55% (Cidex1 OPA, Advanced Sterilization Products). As a positive control for disinfection we used hypochlorite at the manufacturers recommended concentration of 0.87% (Pure Bright Germicidal Ultra Bleach, KIK International). The use of both controls was based on their previous demonstrated efficacy against HPV16 and HPV18 in suspension and carrier tests [33, 34]. All disinfectant products were used according to the manufacturer’s instructions for use.

### Disinfection procedure

Carriers were either disinfected by adding liquid disinfectant or utilizing the automated device. For device disinfection, carriers were transferred onto a rack, which suspended the carriers within the disinfection chamber. A standard cycle using the Antigermix S1 (AS1) device was run in accordance with the manufacturer’s instructions for use. AS1 device disinfects by using Ultraviolet C (UVC) radiation at 253.7nm. The system exposes the target to UVC terminating after the required dose has been delivered on all the surfaces of the probe. A control system adjusts the length of each cycle based on sensor feedback until the required dose is measured. In this way all the surfaces of ultrasound probe receive, at a minimum, the intended germicidal UVC dose during each HLD cycle. For the liquid disinfectants, 2ml of 0.55% OPA or 0.87% hypochlorite was added to the upward facing surface of the carrier so that the liquid formed a droplet covering the entire carrier surface. Carriers were then incubated at room temperature for 12 min (OPA) and 5 min (hypochlorite). Treated viral films were resuspended in 2 ml of cell medium and were collected in a 15ml 100K Amicon filter tube. Two ml of the appropriate neutralizer was added (7% glycine for OPA and a base neutralizer for hypochlorite). The sample was filtered and assayed for infectivity as previously described [33, 34]. All disinfection efficacy tests were conducted in triplicate.

### HPV infectivity assay

Infection was analyzed using a previously described RT-qPCR-based infectivity assay for E1^E4 transcript levels [37]. The E1^E4 spliced transcript was amplified using primers specific for the spliced transcript that do not amplify viral genomic DNA. HPV16 infectivity assays were performed as previously described [34, 37]. HPV18 infectivity assays were performed in the same manner as previously described [34, 37]. Complete viral inactivation was considered achieved when post-disinfection infectivity assays showed equivalent or higher Ct values compared to uninfected controls.

## Results

The use of reusable ultrasound probes is a common protocol in medical practice. Ultrasound probes are used routinely for transvaginal, transrectal, and transesophogeal endocavitary examinations. Coincidentally, these three sites are those that are common sites of HPV infection and cancer. In order to reduce the risk potential for transmission of HPV and other infectious agents healthcare facilities strive to use proper high-level disinfection procedures. Recently, studies have shown that conventional protocols and procedures used to clean and disinfect ultrasound probes have been insufficient in removing residual HPV DNA [25–27]. Studies performed previously by our laboratory demonstrated that disinfectants routinely used by hospitals, including OPA and GTA, were ineffective in killing HPV [33]. Altogether these studies demonstrate a need to identify disinfectants and disinfectant procedures that effective in killing HPV.

Current guidelines from Centers for Disease Control (CDC) have established ultrasound probes as devices used for semi-critical applications (contact with mucous membranes or broken skin) necessitating protocols that use high-level disinfection (HLD) procedures [39]. Therefore, by definition any HLD disinfectant protocol should be able to kill HPV. This is contrary to previous studies in that GTA and OPA are both classified as HLD by the CDC but neither is capable of killing HPV even over extended time periods [33].

In this study, we used the stringent hard surface carrier test method in line with Federal Drug Administration (FDA) guidelines for assessing virucidal efficacy of high-level disinfectants [35, 36]. To test the disinfectants we dried virus in the presence of soil (5% FBS) or hard water (4ppm CaCO_3_) onto carriers made of plastic used in ultrasound transducer construction. We then tested UVC radiation for its efficacy as a disinfectant for HPV16 and HPV18.

**Fig 1:**
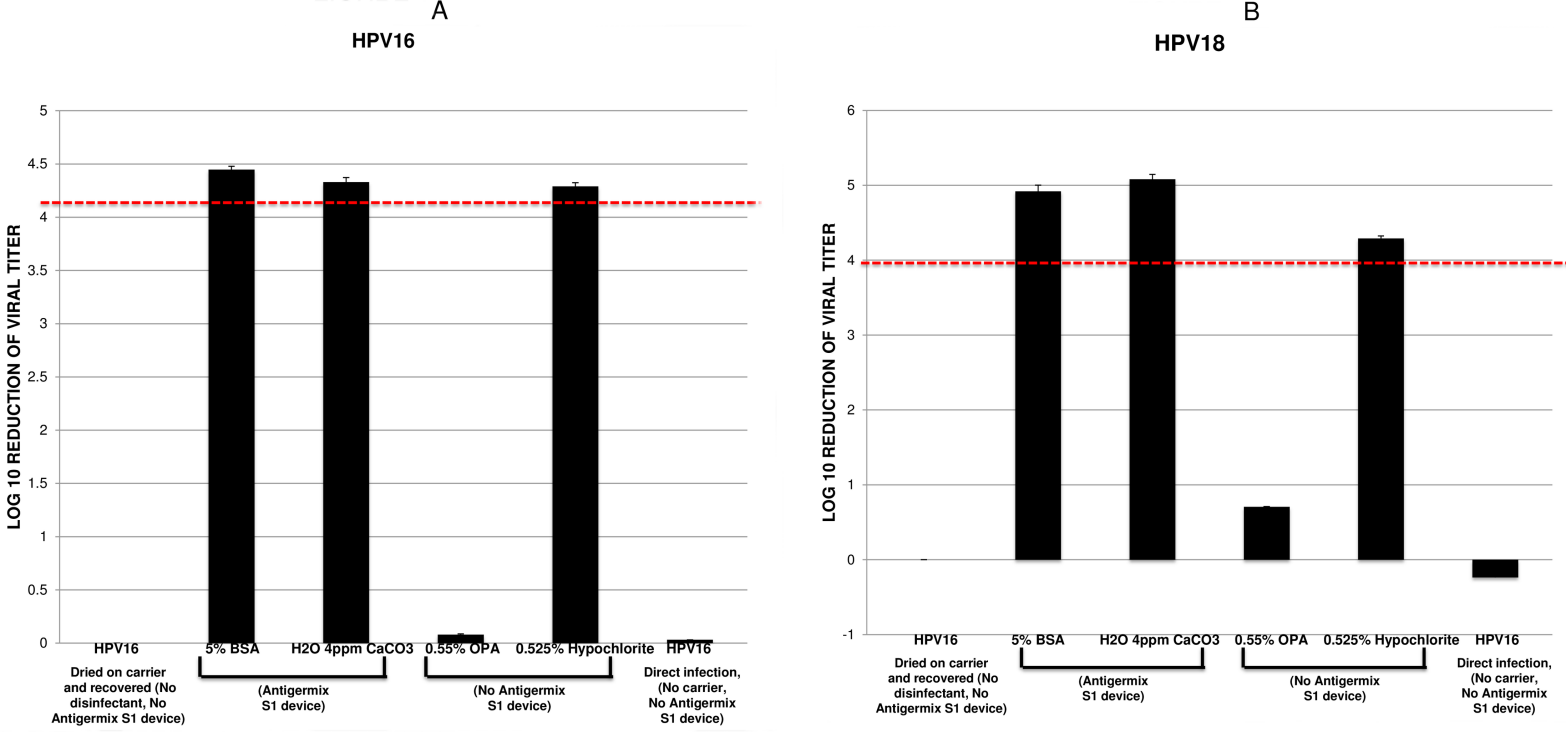
Differing efficacy profiles of disinfectants against HPV. HPV16 (A) or HPV18 (B) virions were subjected to hard surface carrier tests based on the ASTM E1053-11 standard test method against the disinfectants indicated. Virus films were dried onto 50mm diameter ABS carriers in the presence of a 5% fetal bovine serum (soil) and 4ppm CaCO_3_ (hard water) before being disinfected according to the disinfectant or device manufacturer’s instructions. Viral films were assayed for infectivity using a quantitative RT-PCR based method detecting the spliced E1^E4 transcript in infected recipient cells. Post-disinfection infectivity was compared to input to determine log10 reductions. Each efficacy test was conducted in quadruplicate and was paired with a matched neutralization control. Data is expressed as an average of n = 3; error bars indicate standard deviation.

FDA, EPA, and ASTM E1053-11 guidelines state that a hard surface carrier test must be used and show at least a 4 log_10_ reduction in infectivity and achieve complete viral inactivation. In addition, controls for cytotoxicity and neutralization are required to show less than a 0.5 log_10_ reduction, which is clearly demonstrated in our study. In Table 1 we show all of the RT-PCR Ct results that we used to obtain the data in Fig 1.

**Table 1:** Average C_t_ values obtained in this study.

	HPV16		HPV18	
Sample ID	C_t_ Value (Avg)	% Error	C_t_ Value (Avg)	% Error
Virus-Dried on carrier and recovered (No disinfectant, No Antigermix S1 device)	26.89	0.030	24.83	0.079
5% BSA + Virus (Antigermix S1 device)	41.78	0.216	41.25	0.245
H2O + 4ppm CaCO3 + Virus (Antigermix S1 device)	41.06	0.166	41.74	0.130
OPA 0.55% + Virus	28.51	0.174	26.96	0.107
Hyperchlorite 0.87% + Virus	41.94	0.024	41,94	0.024
Virus Infection Only	27.08	0.042	24.60	0.072

Using qRT-PCR we were unable to detect any signal with the uninfected control wells across the primer sets. HPV16 and HPV18, the two most common viruses associated with human cancers, were both highly resistant to OPA showing less than a 1 log_10_ reduction in infectivity (Fig 1). The lack of OPA killing is similar to our previous published results [33, 34]. Hypochlorite, our positive control for HPV killing achieved a >4 log_10_ reduction again in line with previous results [33, 34].

The AS1 UVC system managed to achieve complete inactivation of HPV16 with a >4 log_10_ reduction both with soil (5% BSA) and hard water (4ppm CaCO_3_) included in the assay. The reduction is comparable to that achieved by hypochlorite (Fig 1A). The complete inactivation of HPV18 showed a slightly higher reduction of nearly 5 log_10_ (Fig 1B). Again the inactivation of HPV18 was similar with both soil (5% BSA) and hard water (4ppm CaCO_3_). The differences seen in these values with the same virus type and between virus types reflect different starting titers. These results indicate that the automated high-level disinfection AS1 UVC device was HPV virucidal according to the standard test method efficacy criteria. No cytotoxicity was observed with any of the assays.

## Discussion

In this study we report the results of carrier-based efficacy tests using UVC radiation as a high level disinfectant against native HPV16 and HPV18. Previous studies from our laboratory using both suspension- and carrier-based assays, have shown that HPV16 and HPV18 were greatly to both GTA and OPA [33, 34], two disinfectants categorized as HLD and therefore by definition should be able to kill HPV. In the present study we used OPA as a negative control for HPV killing. Our results demonstrating that OPA and GTA, two commonly used hospital sterilants were incapable of killing HPV was cause for concern. Couple this with additional studies by others showing that hospital protocols for disinfecting endocavitary probes were not effective in removing HPV contamination [25–27]. Recommended guidelines as presently constituted create a potential risk of unintended exposure of a patient to HPV. At this time there are no studies that have measured the prospect of iatrogenic transmission of HPV. Iatrogenic transmission of HPV could take years even decades to develop symptoms making it difficult to trace the source of exposure. Theoretical transmission risk has been the basis for guideline recommendations [40].

Mycobacterium species have displayed resistance to the aldehydes GTA and OPA with transmission from patient to patient as an outcome [41]. GTA and and OPA and similar aldehydes use a mechanism of action that crosslinks reactive sites on proteins like exposed primary amines (i.e. lysines) and thiols (i.e. cysteines) [42]. The presence of aldehyde reactive sites on the surface of HPV capsids is under investigation.

As part of our continuing investigations to better understand the requirements for HPV directed disinfection we have demonstrated the efficacy of using UVC radiation as a potential platform to deactivate HPV on re-usable semi-critical hospital devices. UVC radiation has been shown effective in eliminating bacteria, including *Clostridium difficile* spores, on contaminated surfaces in a hospital room [43] and is becoming a common part of a healthcare facility’s arsenal to prevent nosocomial infections. UVC radiation is at 253.7nm a level that is absorbed by DNA preferentially over proteins, suggesting that it could deactivate HPV by causing lesions like pyrimidine dimers in the viral genome subsequently interfering with replication and transcription.

We have previously shown that hypochlorite is effective in deactivating HPV [33] and use it as a positive control for killing HPV in our studies [34]. However, hypochlorite is not considered a high level disinfectant and is not compatible with the materials used to make semi-critical devices. Our studies using UVC radiation were performed according to standards meant to simulate worst-case clinical conditions including, high viral titer, high protein soil, and hard water in accordance with FDA requirements. We also used in this study and in previous studies, native infectious HPV virus produced in differentiating epithelium mimicking the in vivo situation [37, 44]. We have before demonstrated that quasivirions, formed by the spontaneous assembly from artificially expressed proteins and DNA, have a significant different resistance profile to common disinfectants when compared to native HPV virions [33]. This study shows that efficacy claims need to be supported by data using native virions as quasivirions have a different efficacy profile and therefore cannot serve as a proper surrogate. Additionally, other viruses such as the polyomavirus SV40 has been used by some claiming it to be suitable surrogate for HPV. Polyomaviruses are a different family viruses and until there are comparisons done side-by-side with native HPV directly, we should be cautious in using SV40 or any other surrogate’s resistance profile as an indicator for HPV efficacy. Additionally, chemistries, contact times, dosage, and other test conditions should not be generalized.

HPV is a major viral pathogen, as discussed, causing more diseases than just cervical cancer. Much work has been done identifying these viruses as the most common sexually transmitted disease. Here, we educate that HPVs are very stable viruses, able to survive on fomites and surfaces for days. HPV is available to be transmitted through non-sexual means, either by way of mother to child, fomites, self-inoculation, nosocomial, or iatrogenic infections. It is also possible that other less documented forms of transmission of HPV infection may be possible. However, more research is necessary to determine the rate and consequences of nonsexually transmitted HPV. Given the well understood cancer-risk associated with HPV infection and patient expectation that their safety is paramount, healthcare providers need to give careful consideration to their disinfection practices and how they manage risk in regard to HPV in relation to endocavity ultrasound probe reprocessing.

## Supporting information

S1 File Supporting information file UVC Radiation. (DOCX)
