# Supplementary material for: UVC radiation as an effective disinfectant method to inactivate human papillomaviruses
Source: PLoS One. 2017 Oct 31;12(10):e0187377. doi: 10.1371/journal.pone.0187377 (PMC5663507; doi:10.1371/journal.pone.0187377)
Supplement: S1 File — (DOCX) [file pone.0187377.s001.docx]

**
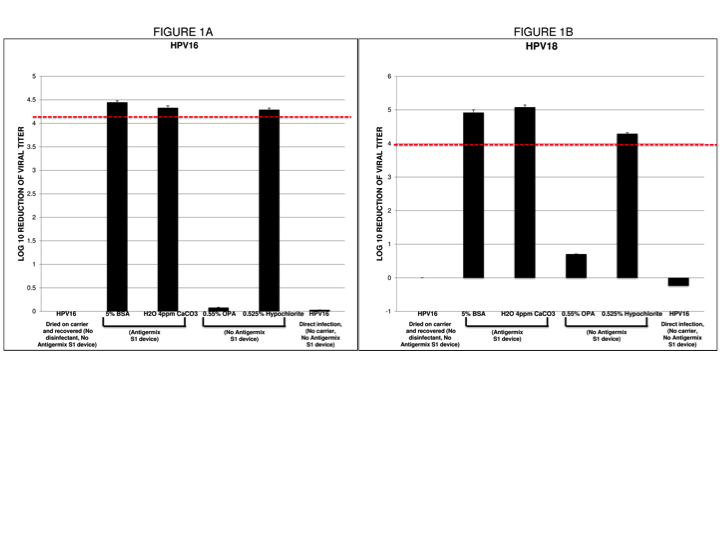
Supporting Information**

**Fig. 1. Differing efficacy profiles of disinfectants against HPV.** HPV16 (A) or HPV18 (B) virions were subjected to hard surface carrier tests based on the ASTM E1053-11 standard test method against the disinfectants indicated. Virus films were dried onto 50mm diameter ABS carriers in the presence of a 5% fetal bovine serum (soil) and 4ppm CaCO_3_ (hard water) before being disinfected according to the disinfectant or device manufacturer’s instructions. Viral films were assayed for infectivity using a quantitative RT-PCR based method detecting the spliced E1^E4 transcript in infected recipient cells. Post-disinfection infectivity was compared to input to determine log10 reductions. Each efficacy test was conducted in quadruplicate and was paired with a matched neutralization control. Data is expressed as an average of n=3; error bars indicate standard deviation.

**Table 1. Average C_t_ values obtained in this study.**

|  | **HPV16** | | **HPV18** | |
| --- | --- | --- | --- | --- |
| **Sample ID** | **C_t_ Value (Avg)** | **% Error** | **C_t_ Value (Avg)** | **% Error** |
| Virus-Dried on carrier and recovered (No disinfectant, No Antigermix S1 device) | 26.89 | 0.030 | 24.83 | 0.079 |
| 5% BSA + Virus (Antigermix S1 device) | 41.78 | 0.216 | 41.25 | 0.245 |
| H2O + 4ppm CaCO3 + Virus (Antigermix S1 device) | 41.06 | 0.166 | 41.74 | 0.130 |
| OPA 0.55% + Virus | 28.51 | 0.174 | 26.96 | 0.107 |
| Hyperchlorite 0.87% + Virus | 41.94 | 0.024 | 41,94 | 0.024 |
| Virus Infection Only | 27.08 | 0.042 | 24.60 | 0.072 |

Using qRT-PCR we were unable to detect any signal with the uninfected control wells across the primer sets. HPV16 and HPV18, the two most common viruses associated with human cancers, were both highly resistant to OPA showing less than a 1 log_10_ reduction in infectivity (Fig. 1). The lack of OPA killing is similar to our previous published results [33, 34]. Hypochlorite, our positive control for HPV killing achieved a >4 log_10_ reduction again in line with previous results [33, 34].

The AS1 UVC system managed to achieve complete inactivation of HPV16 with a >4 log_10_ reduction both with soil (5% BSA) and hard water (4ppm CaCO_3_) included in the assay. The reduction is comparable to that achieved by hypochlorite (Fig 1A). The complete inactivation of HPV18 showed a slightly higher reduction of nearly 5 log_10_ (Fig. 1B). Again the inactivation of HPV18 was similar with both soil (5% BSA) and hard water (4ppm CaCO_3_). The differences seen in these values with the same virus type and between virus types reflect different starting titers. These results indicate that the automated high-level disinfection AS1 UVC device was HPV virucidal according to the standard test method efficacy criteria. No cytotoxicity was observed with any of the assays.
